# Impact of Nutrient Restriction on the Structure of *Listeria monocytogenes* Biofilm Grown in a Microfluidic System

**DOI:** 10.3389/fmicb.2017.00864

**Published:** 2017-05-17

**Authors:** Tamazight Cherifi, Mario Jacques, Sylvain Quessy, Philippe Fravalo

**Affiliations:** ^1^Chaire de recherche en salubrité des viandes, Faculté de médecine vétérinaire, Université de MontréalSaint-Hyacinthe, QC, Canada; ^2^Groupe de Recherche sur les Maladies Infectieuses du Porc, Faculté de médecine vétérinaire, Université de MontréalSaint-Hyacinthe, QC, Canada; ^3^Centre de Recherche en Infectiologie Porcine et Avicole, Faculté de médecine vétérinaire, Université de MontréalSaint-Hyacinthe, QC, Canada; ^4^Réseau canadien de recherche sur la mammite bovine et la qualité du lait, Faculté de médecine vétérinaire, Université de MontréalSaint-Hyacinthe, QC, Canada

**Keywords:** limited nutrients, extracellular DNA, *Listeria monocytogenes*, biofilm structure, microfluidic system

## Abstract

Biofilm formation by the pathogen *Listeria monocytogenes* is a major concern in food industries. The aim of this work was to elucidate the effect of nutrient limitation on both biofilm architecture and on the viability of the bacteria in microfluidic growth conditions. Biofilm formation by two *L. monocytogenes* strains was performed in a rich medium (BHI) and in a 10-fold diluted BHI (BHI/10) at 30°C for 24 h by using both static conditions and the microfluidic system Bioflux. In dynamic conditions, biofilms grown in rich and poor medium showed significant differences as well in structure and in the resulting biovolume. In BHI/10, biofilm was organized in a knitted network where cells formed long chains, whereas in the rich medium, the observed structure was homogeneous cellular multilayers. Biofilm biovolume production in BHI/10 was significantly higher than in BHI in these dynamic conditions. Interestingly, biovolume of dead cells in biofilms formed under limited nutrient conditions (BHI/10) was significantly higher than in biofilms formed in the BHI medium. In the other hand, in static conditions, biofilm is organized in a multilayer cells and dispersed cells in a rich medium BHI and poor medium BHI/10 respectively. There was significantly more biomass in the rich medium compared to BHI/10 but no difference was noted in the dead/damaged subpopulation showing how *L. monocytogenes* biofilm could be affected by the growth conditions. This work demonstrated that nutrient concentration affects biofilm structure and the proportion of dead cells in biofilms under microfluidic condition. Our study also showed that limited nutrients play an important role in the structural stability of *L. monocytogenes* biofilm by enhancing cell death and liberating extracellular DNA.

## Introduction

*Listeria monocytogenes* is a pathogenic foodborne bacterium that causes listeriosis. Biofilm formation by *L. monocytogenes* is a major concern in the food industry because it generates recurring risks of ready-to-eat food contamination notably by enhancing resistance to disinfection treatments during sanitation procedures (Di Bonaventura et al., 2008). Therefore, a better understanding of the mechanisms and factors influencing biofilm formation would help in its prevention. *L. monocytogenes* biofilm formation is affected by many conditions like surface type, temperature, and growth medium (Di Bonaventura et al., 2008; Combrouse et al., 2013). In previous studies, biofilm production in rich and poor media were compared to assess the effects of nutrient concentration. Results have been ambiguous, with some authors concluding that biofilm production is enhanced in nutrient-poor medium (Zhou et al., 2012; Combrouse et al., 2013; Kadam et al., 2013) and others finding that in such conditions *L. monocytogenes* produces less biofilm (Stepanovic et al., 2004; Harvey et al., 2007; Guilbaud et al., 2015). All of these studies describe the effect of nutrient concentration on biomass production. However, there is no information, to the best of our knowledge, about how limited nutrients affect the structure of the biofilm produced by *L. monocytogenes*.

Most authors who have described the structure of *L. monocytogenes* biofilm have observed a honeycomb structure (Marsh et al., 2003; Guilbaud et al., 2015), however Rieu et al. (2008), using diluted media tryptic soy broth (TSB), described ball-shaped structures with elongated chain cells when grown in dynamic conditions (flow cell) and multilayer cell structures when grown in static conditions. These authors proposed this was due to the differences between these two growth conditions and argued that in flowing conditions bacteria are submitted to shear forces and flow. Previous studies on other biofilm forming bacteria underlined that different flow conditions can change biofilm structure. *Pseudomonas aeruginosa*, forms biofilm in streamlined patches and ripple-like wave structures in turbulent flow and monolayers interspersed with small colonies in laminar flow (Purevdorj et al., 2002). For this bacterium, along with an increase in nutrient concentration (Carbon and Nitrogen), the biomass and thickness of biofilm increased as well and the morphology changed with flow conditions (Stoodley et al., 1998).

Most of the available information on the establishment of *L. monocytogenes* biofilm structure and biomass was generated mainly by two different methods: microtiter plates (Sela et al., 2006) or culture chambers (Pilchová et al., 2014) for static conditions. A few other authors used flow cells for dynamic conditions (Rieu et al., 2008; Harmsen et al., 2010). Recently, a new method for assessing biofilm formation was developed that provides microfluidic growth conditions (Meyer et al., 2011). Based on microfluidic conditions and microscale flow, this system enables the accurate control of the microenvironment, like temperature and stable fluid flow around the biofilm (Janakiraman et al., 2009; Kim et al., 2012), while other macroscopic dynamic systems (drip flow, flow-cell) involve the use of large flow cells that make it difficult to control the microenvironment around the biofilm (Janakiraman et al., 2009). This microfluidic system has been used to study the biofilm of some species like *P. aeruginosa* and *Staphylococcus aureus* (De Rienzo et al., 2016) but it has not been used to study *L. monocytogenes* biofilm yet. In this work, the microfluidic system was used along with static methods to study the effect of nutrient concentration on *L. monocytogenes* biofilm structure and assess how limited nutrients affect the stability of a knitted network-like structure in microfluidic conditions. This study showed that limited nutrient conditions enhanced a knitted-like structure formation composed of cell chains in *L. monocytogenes* biofilm and that this structure depends directly on extracellular DNA (e-DNA), which seems to be involved in the stabilization of *L. monocytogenes* biofilm structure in microfluidic conditions.

## Materials and methods

### Bacteria and strains

Strains; Lm76 and Lm132, which belong to serotype 1/2a and serotype 1/2b respectively, were isolated from pork slaughterhouses and cutting facilities after sanitation procedures. Strains were conserved in a Brucella broth and 15% glycerol at −80°C. Before all experimentations, strains were streaked on blood agar and incubated overnight at 37°C.

### Biofilm formation in a static microtiter plate assays

An overnight culture of *L. monocytogenes* strains was used for biofilm production in 96-well plates (Costar® 3370; Corning, NY, USA). One hundred microliters of an overnight culture in TSBYE, to an optical density (OD600) of 1, at 37°C was added to 10 ml of fresh BHI (Becton and Dickinsen, Franklin Lakes, NJ, USA) and 100 μl of the inoculated BHI was distributed into each well. Microplates were covered and the sides were surrounded by parafilm to avoid evaporation and incubated at 30°C for 24 h.

### Biofilm formation using the microfluidic system

The BioFlux 200 system with 48-well plates (Fluxion biosciences, South San Francisco, California, USA) was used for biofilm formation. The protocol developed by Benoit et al. (2010) and Tremblay et al. (2015) for other species was adapted for *L. monocytogenes* biofilm formation. Important parameters such as shear stress of 1 or 0.5 dyn/cm^2^, which represents the pressure applied to the medium [1 Pascal (Pa) = 10 dyn/cm^2^], adhesion time (2 or 4 h), and concentration of the inoculum correspond to an optical density of 0.25, 0.5, or 1 were tested for biofilm production in the microfluidic system. Finally, two concentrations of the medium—a full strength BHI and a 10-fold BHI (BHI/10)—were retained for a comparison in the following conditions: 1.5 ml of an overnight culture (OD_600_ = 1) of the Lm132 and Lm76 strains was centrifuged and resuspended in a fresh pre-warmed medium and 100 μl of these cultures was added to the output well of the BioFlux instrument then injected at 0.05 Pa of shear stress for 30 s into the microfluidic channels, which had been wetted with a fresh pre-warmed BHI medium. Plates were incubated at 30°C for 4 h without flow to allow bacteria attachment to the surface of the chambers, and the wells were equilibrated by adding 100 μl of pre-warmed sterile medium in the input wells.

After 4 h, the input wells were filled with 1.25 ml of pre-warmed BHI or BHI diluted with demineralized water at 1/10 (v/v) and the 48-well plates were incubated at 30°C. The shear stress was then applied at 0.05 Pa for 24 h and the flow was run at 50 μl/h. Biofilm growth was monitored by taking pictures every 10 min for 24 h using an inverted fluorescence microscope (Olympus CKX41) equipped with a 10X objective and a digital camera (Retiga EX; Q Imaging) and a time lapse video was created from these pictures.

### Image processing

Biofilms were stained with crystal violet 0.1 % (w/v) and live/dead dye: Syto 9, a green cell permeant dye of acid nucleic (to stain live cells) and Propidium iodide, a red impermeant nucleic acid dye to stain damaged or dead cells (Molecular probes, Eugene, OR, USA). The staining with the two markers was performed as follows: After 24 h of biofilm growth, the flow was stopped and the residual medium and the effluent were removed completely from the inlet and outlet wells, 100 μl of CV or live/dead was added in the inlet well, and the flow was activated for 10 min for CV and 20 min for live/dead. Finally, the biofilm was washed by adding 100 μl of fresh BHI medium for 10 min under the flow. For biofilm stained with crystal violet, images were taken with an inverted fluorescent microscope equipped with a 40X objective (CKX41; Olympus, Markham, ON, Canada); image acquisition of the biofilm stained with live/dead dye was performed with a Confocal Laser Scanning Microscope (CLSM; Olympus FV1000 IX81) equipped with a 40X objective and 488 nm of argon and 543 of helium/Neon lasers. The green fluorescence of Syto9 was excited at 488 nm and the emission fluorescence was collected between 500 and 555 nm, the red fluorescence of PI was excited at 543 nm and the fluorescence emitted was collected between 555 and 625 nm. Three dimensional images (3D) were constructed for each strain and each condition by taking 13–25 image layers from the surface to the bottom of the biofilm; the Z-distance was kept within the same range between each sample. The biovolume of live and dead or damaged populations was calculated separately to estimate the respective biomass. Briefly, from the reconstructed 3D images of each dead and live biomass, an isoimage, which is an image that computes and draws a surface within a volumetric data field, on a 3D image, was created and the volume was calculated considering the height and the surface of the 3D image. The sum of both populations corresponds to the total biofilm. Biovolume calculation was repeated independently on different days.

All image analyses and biovolume calculations were performed with Image-Pro software (version 9.0; Media Cybernetics, Inc., Bethesda, MD, USA).

### DNase treatment in microfluidic conditions

Biofilm grown in microfluidic conditions was submitted to DNase treatment; growth conditions were the same as described above. At 24 h, biofilms formed in full strength and diluted BHI were subjected to DNase I treatment (Promega), prepared in 150 mM of NaCl and 1 mM CaCl_2_ solution and added into the fresh mediums for a final concentration of 100 μg/ml (w/v). The flow was activated under the same conditions the biofilm was grown (0.05 Pa at 30°C for 18 h). After 18 h, the biofilm was stained with live/dead dyes and image analysis was performed with CLSM.

### Statistical analysis

Data were compared with a Student *t*-test after log transformation of the dead, live and total biomasses, *p* < 0.05 was considered statistically significant.

## Results

### Concentration of nutrients affect biofilm structure

**Figure 1:**
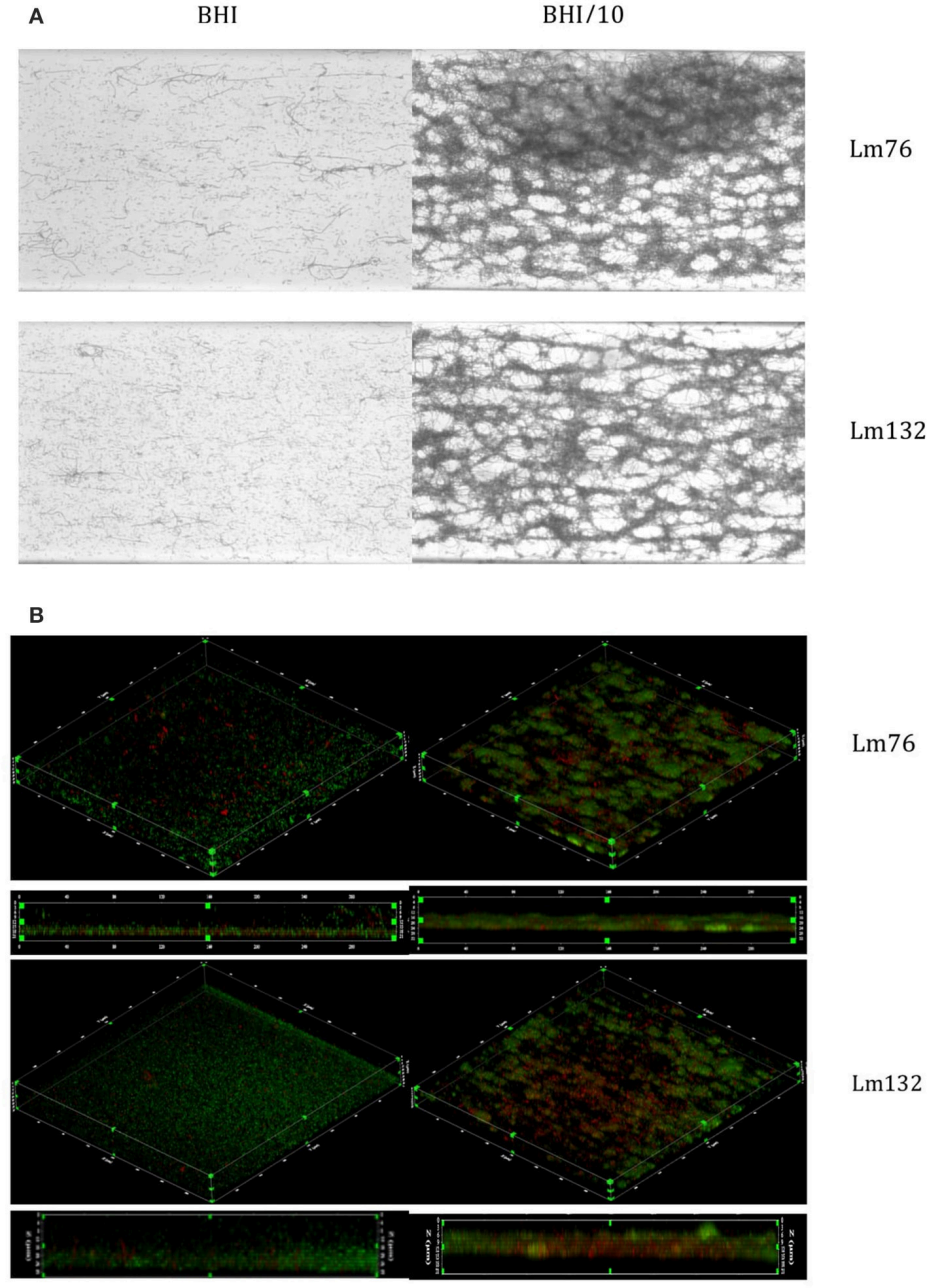
**Biofilm visualization of two strains of ***Listeria monocytogenes***—Lm76 and Lm132—after 24 h of incubation at 30°C in microfluidic conditions**. Biofilm was grown in BHI medium (left images) and BHI/10 (right images); **(A)** Biofilm stained with Crystal violet 0.1% **(B)** 3D reconstruction of *L. monocytogenes* biofilm stained with live/dead; Syto9 showing live cells in green and Propidium iodide showing dead/damaged cells and e-DNA in red **(B)**.

**Figure 2:**
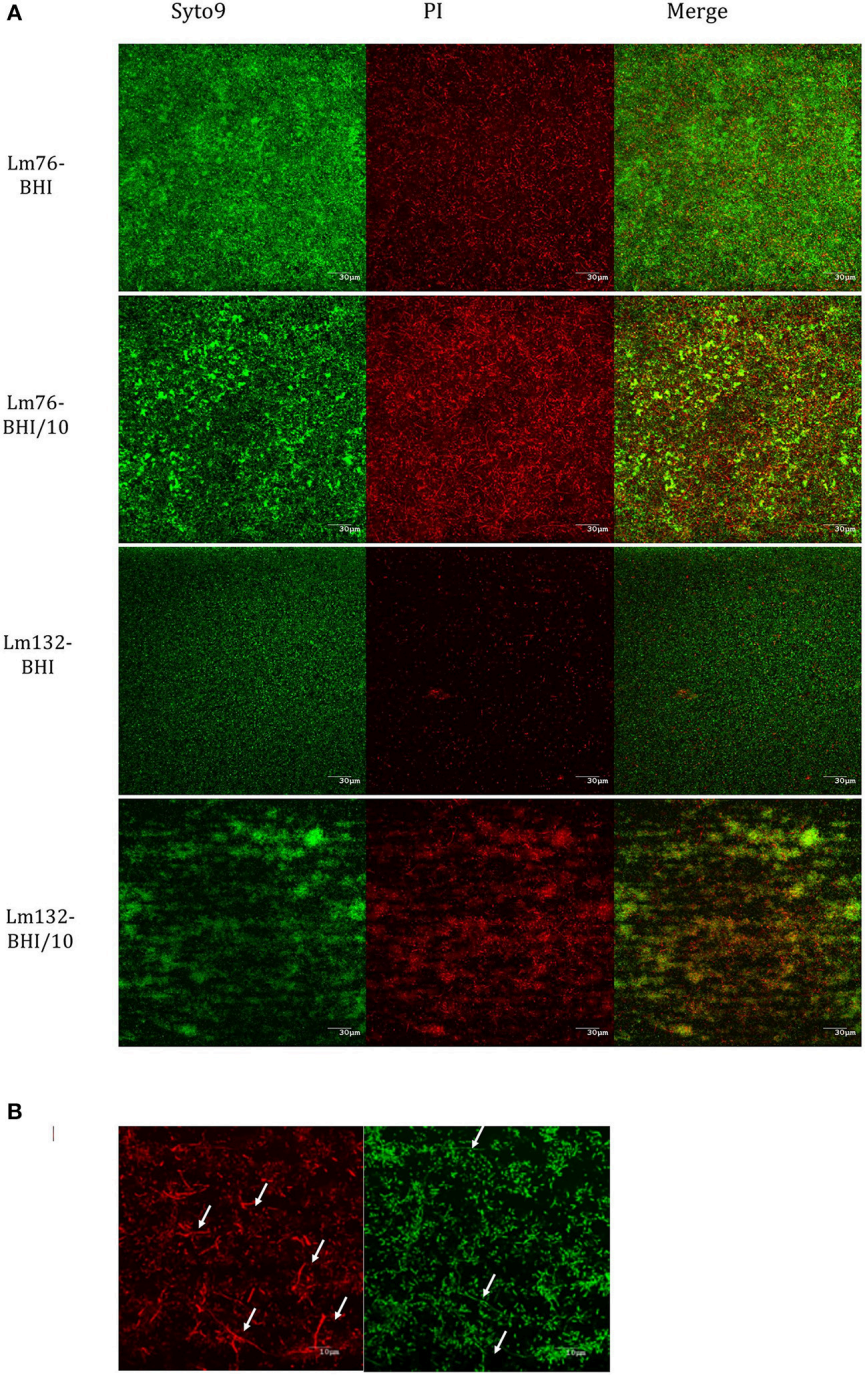
**Composition of dead and live cells in the biofilm of Lm76 and Lm132 strains of *L. monocytogenes* formed in rich medium BHI and diluted medium BHI/10 for 24 h; (A)** individual visualization of live population of the biofilm (left images), dead population (middle image) and the merge of the two images which represents the compilation of all images taken from the top of the biofilm to the bottom and corresponds to the total biomass formed in the biofilm (right image). **(B)** higher magnification (taken with a 100X objective) of Lm76 biofilm grown in the diluted medium BHI/10 showing dead (right image) and live (left image) biomass organization; arrows show filaments mostly present in dead biomass in the biofilm.

Biofilm formed by both Lm76 and Lm132 strains, as shown by crystal violet staining after 24 h of flow at 30°C, revealed different morphologies depending on the concentration of the medium. In the rich medium (BHI), the biofilm structure appeared uniformly organized, showing stacked cellular layers and some dispersed filaments with both the Lm76 and Lm132 strains (Figure 1A). On the contrary, when subjected to the 10-fold diluted medium (BHI/10), both the Lm76 and Lm132 biofilms showed structures that were completely different from those seen in biofilm grown in a rich medium; organized cells in a condensed network formed by entangled filaments that produced clear holes and ball-shaped structures called microcolonies were observed (Figure 1A). Three dimensional images revealed multilayered cellular structures for both strains (Figure 1B). In the rich medium there was a regular distribution of live (green) and dead cells (red) over the chambers (left images, Figure 1B) whereas in a 10-fold diluted medium (BHI/10), biofilms were formed by microcolonies which were composed of live cells (green fluorescent) and dead cells (red fluorescent) between and underneath these so-called microcolonies as shown in the live/dead image edge (right images, Figure 1B). In biofilms of both strains formed in rich media rather than poor, this interaction between live and dead cells that produced the knitted network-like structure does not seems to occur (Figure 1B). It is interesting to note that when grown in a rich medium, the two strains (but particularly Lm76) were able to form filaments as shown during an overnight incubation but, in the case of later phase biofilm development, the observed filaments detached from the surface and were carried off by the flow as demonstrated in the overnight video of Lm 76 biofilm in the Video S1. On the contrary, in the poor medium, the filaments were involved in the structure and for the same duration did not show any sign of apparent disturbance as observed in the Lm76 strain biofilm growth in the Video S2. Interestingly, microcolonies were represented mostly by live cells (Figure 2A) and PI stained mostly the filaments which formed a knitted network, as demonstrated in images taken with a 100X objective of Lm76 biofilm (Figure 2B).

**Figure 3:**
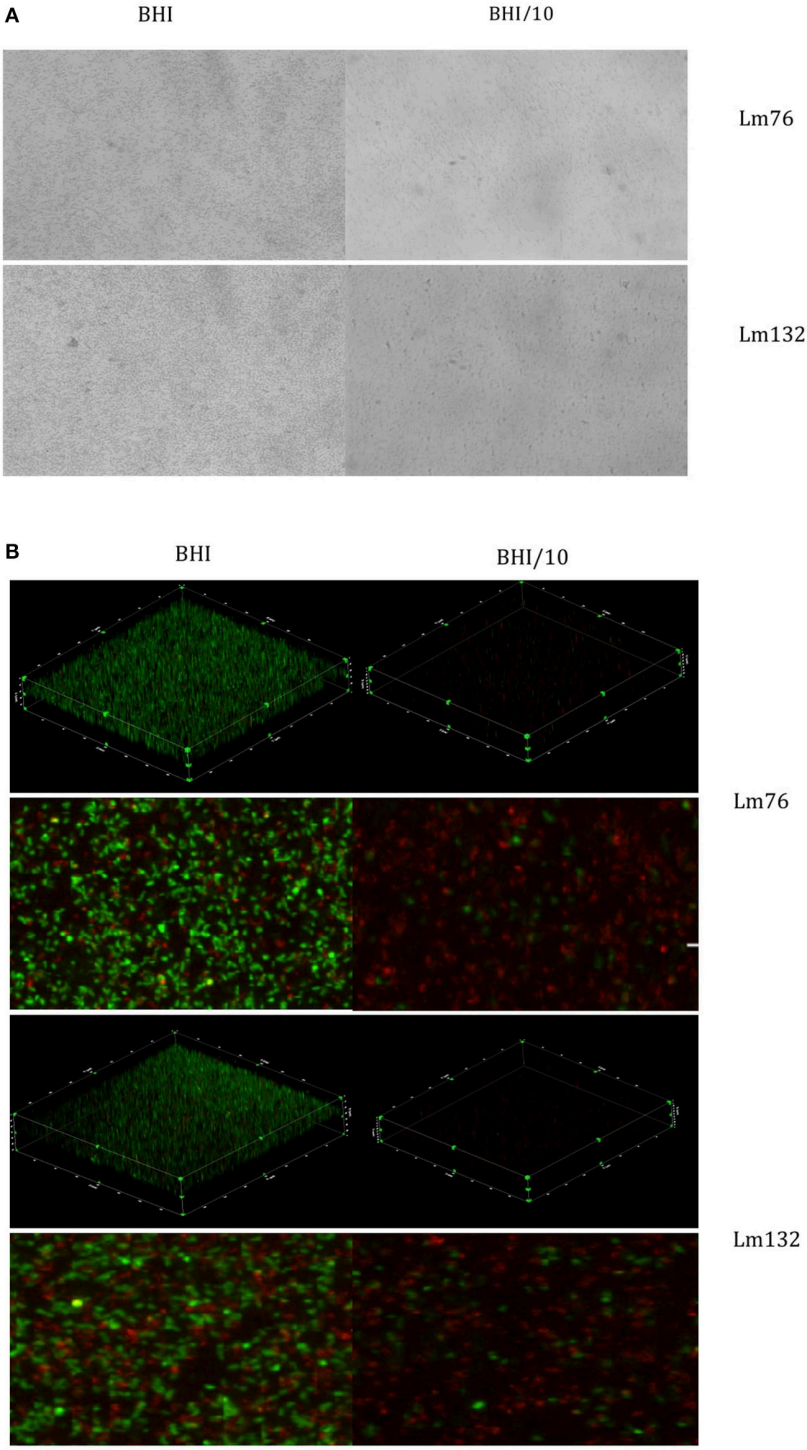
**Biofilm visualization of two strains of ***Listeria monocytogenes***: Lm76 and Lm132 after 24 h of incubation at 30°C static conditions**. Biofilm was grown in BHI medium (right images) and BHI/10 (left images); **(A)** Biofilm stained with Crystal violet 0.1% **(B)** 3D reconstruction, zoomed images of a view from above of *L. monocytogenes* biofilm stained with live/dead; Syto9 showing live cells in green and Propidium Iodide showing dead/damaged cells and e-DNA in red.

To determine if this phenomenon also occurs in other growth conditions, biofilm formation of these two strains was tested in static conditions, which is still the most common method used to study biofilm formation (Djordjevic et al., 2002; Cole et al., 2004; Stepanovic et al., 2004). Surprisingly, biofilms stained with crystal violet were formed of multilayered cells regardless of the nutrient concentration (rich or poor medium) for both Lm76 and Lm132 (Figure 3A).

The live/dead dye used to stain the biofilm in static conditions and 3D images confirmed the presence of multilayered cells for the two strains in both tested media (BHI and BHI/10) even if in BHI/10 medium it appeared there was limited growth and low biomass present and cells were dispersed over the surface (Figure 3B). The biofilm was formed mainly by live cells in the rich medium BHI whereas in BHI/10, dead or stressed biomass was the largest population present (Figure 3B).

### Low nutrient concentration enhances biofilm formation by cell death

**Figure 4:**
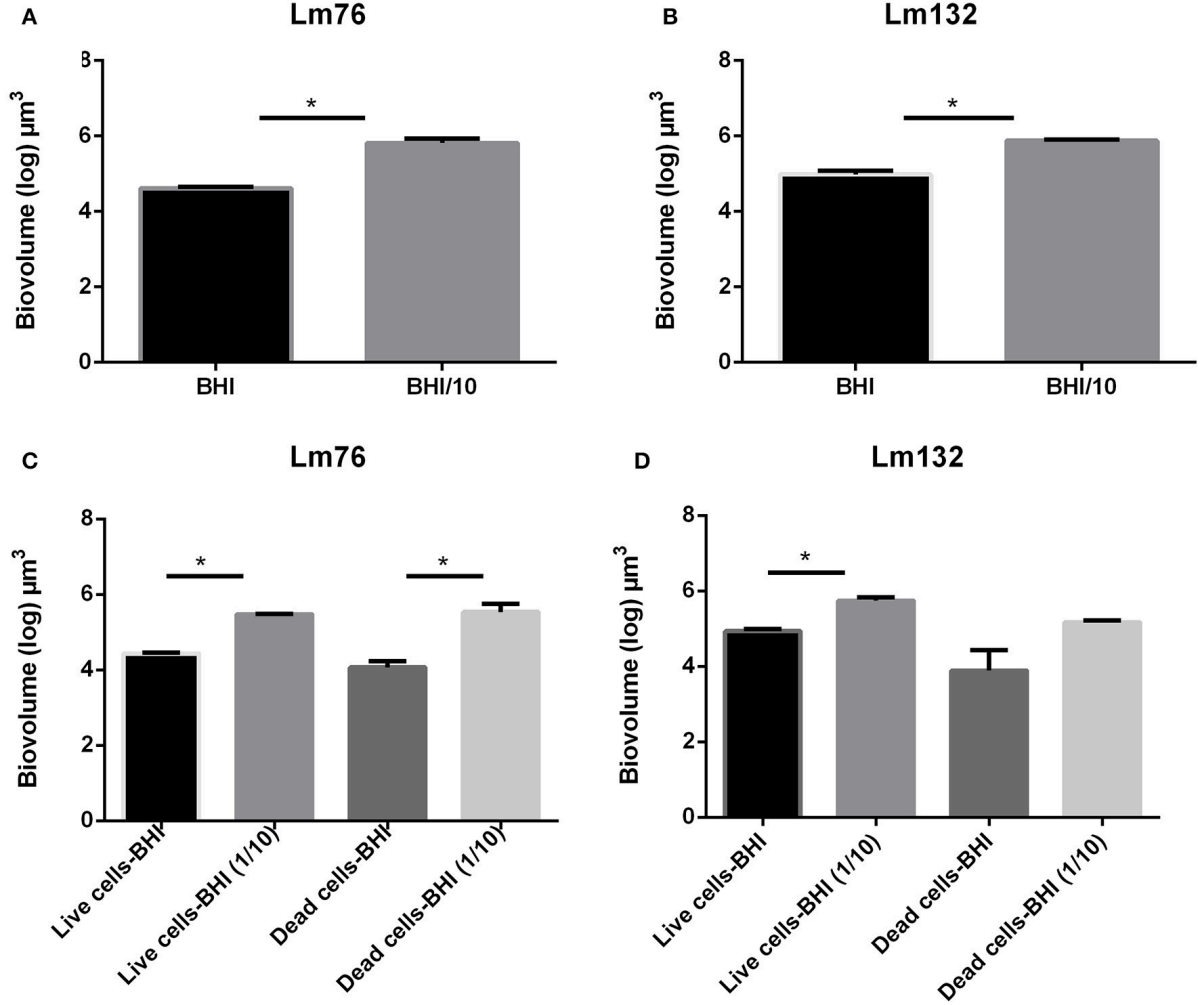
**Biovolume calculation of biofilm formed in microfluidic conditions by ***Listeria monocytogenes*** Lm76 and Lm132 strains grown in a rich medium BHI and poor medium BHI/10 for 24 h at 30°C; (A,B)** Total biovolume of biofilm formation by Lm76 and Lm132 strains respectively which corresponds to the some of the live and dead biomass in each biofilm; **(C,D)** Biovolume of live (green cells) and dead (red cells) biomasses in Lm76 and Lm132 biofilm, respectively. ^*^P < 0.05.

To evaluate the biomass in the biofilm, the biovolume of live and dead biomasses extracted from the 3D images obtained from the CLSM was calculated. In microfluidic condition, differences in biovolume depending on the concentration of the medium was noted; the biofilm volume in a poor medium (BHI/10) was larger than in a rich medium for both Lm76 and Lm 132 strains (Figure 4A) and (Figure 4B) respectively. The biomass of dead cells depending on nutrient concentration conditions, was significantly higher in a poor medium (BHI/10) than in a rich medium (BHI; Figure 4C) for Lm76. Likewise, results showed that with the Lm132 strain there were more dead cell amounts in the BHI/10 medium than in BHI; however, differences did not reach statistical significance (Figure 4D).

**Figure 5:**
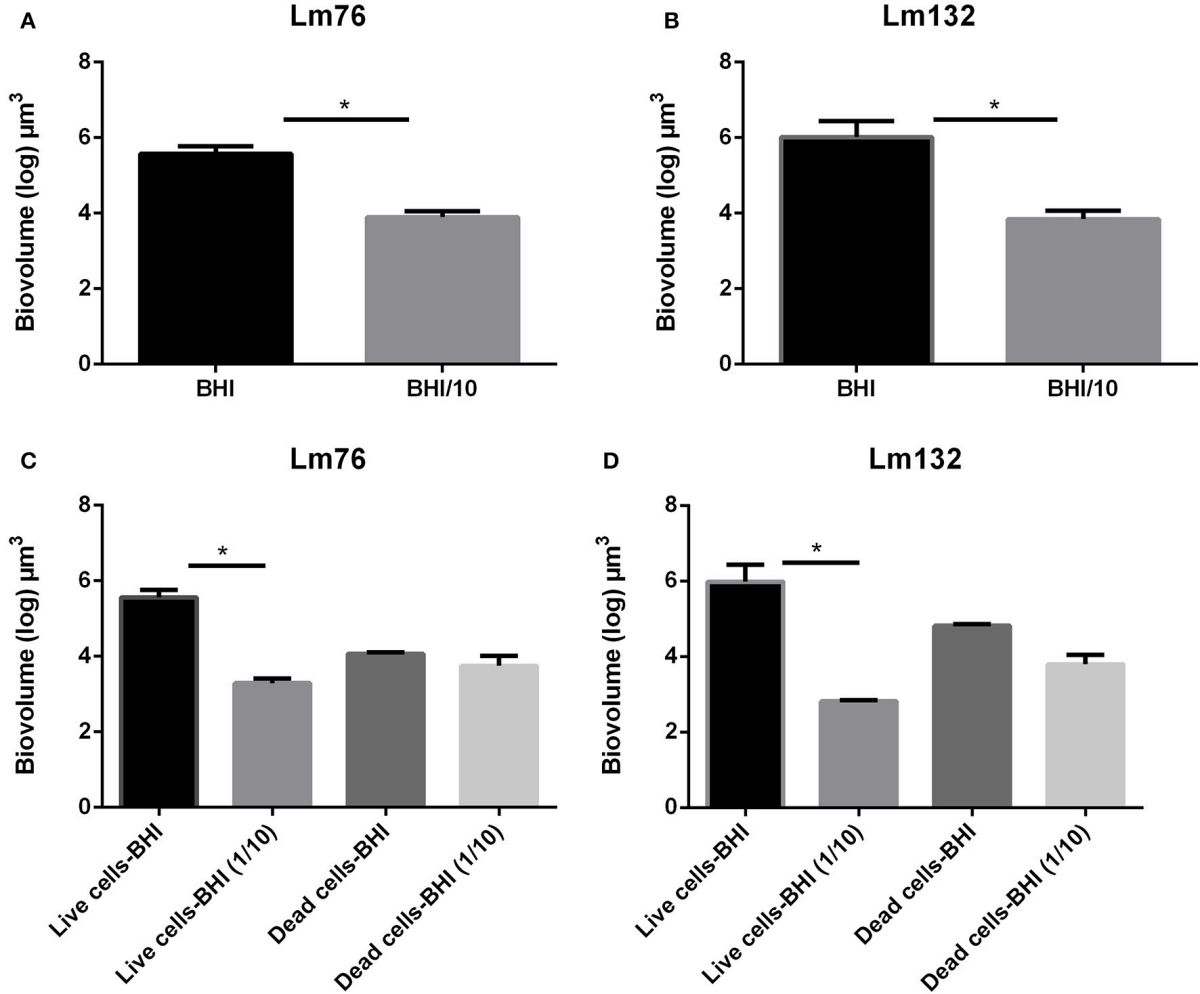
**Biovolume calculation of biofilm formed in static conditions by ***Listeria monocytogenes*** Lm76 and Lm132 strains grown in a rich medium BHI and poor medium BHI/10 for 24 h at 30°C; (A,B)** Total biovolume of biofilm formation by Lm76 and Lm132 strains respectively which corresponds to some of the live and dead biomass in each biofilm; **(C,D)** Biovolume of live (green cells) and dead (red cells) biomasses in Lm76 and Lm132 biofilm respectively. ^*^P < 0.05.

In static conditions, the total biovolume was higher in rich medium than in poor medium in both strains (Figure 5A) and (Figure 5B) and the value of live cell biomass in rich medium was significantly higher than in poor medium (Figure 5C) and (Figure 5D), whereas there were no significant differences in dead biomass between rich and poor medium for the Lm76 strains (Figure 5C) and Lm132 (Figure 5D) although in rich medium there was more dead biomass.

### No e-DNA, no knitted network structure

**Figure 6:**
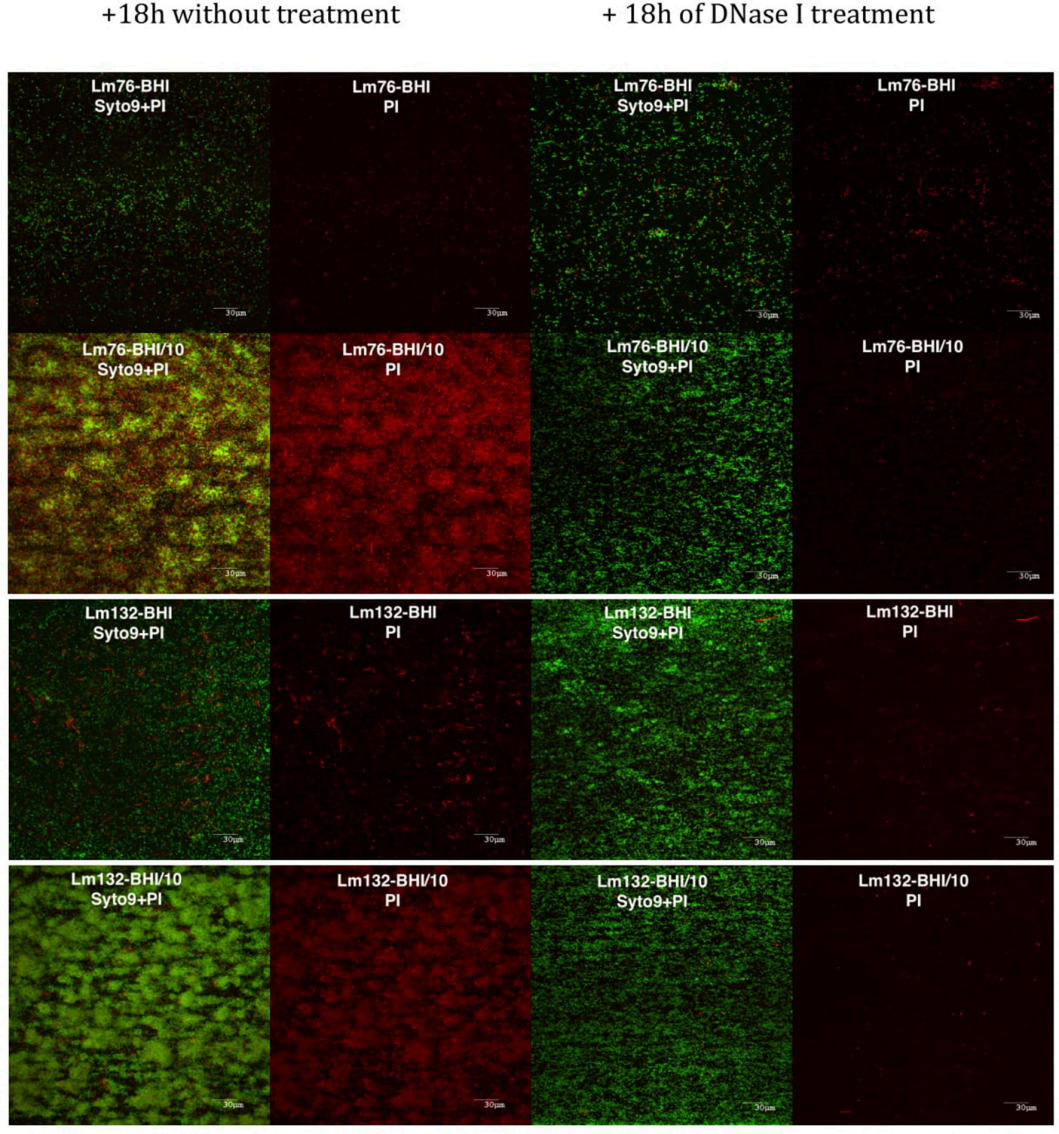
**Biofilm of Lm76 and Lm132 grown under BHI or BHI/10 medium as a control (right images) and BHI or BHI/10 containing 100 μg/ml of DNase I (left images)**. All biofilms were stained with Syto9 and PI dyes after 18 h with DNase treatment or without treatment. The images show, from the left to the right, biofilm stained with Syto9 and PI without treatment (the two left images) and biofilm stained with Syto9 and PI after DNase treatment to show the decrease of e-DNA and dead/damaged biomass.

As described above, the presence of a greater dead cell biomass in poor medium, under microfluidic conditions, suggests that e-DNA could be involved in the knitted network-like structure stabilization of *L. monocytogenes* biofilm. This prompted us to test the effect of DNase I on biofilm structure. After 18 h of treatment with DNase I, biofilm structure grown in diluted BHI medium changed drastically, completely losing its filamentous structure (Figure 6). The effect of DNase on *L. monocytogenes* biofilm was spectacular; upon turning the flow on, all the biofilm detached (Figure 6), which can be seen in the 18 h video provided in Videos S3, S4. At 400X magnification, small live cellular sticks could be observed but no more filaments were observed any time after DNase treatment. More interestingly, the dead/damaged biomass almost totally disappeared from the biofilm, unlike with the untreated biofilm (Figure 6B). It is interesting to note that after a few seconds of DNase treatment, almost all biofilm detached in the poor and rich mediums but after 18 h live biomass could be observed under DNase treatment (Figure 6).

## Discussion

This study demonstrated that limited nutrients and e-DNA are essential for the establishment of a knitted-like structure in *L. monocytogenes* biofilm in microfluidic conditions. It was clearly evident that a BHI medium and a 10-fold diluted BHI provided completely different results, particularly regarding the structure of *L. monocytogenes* biofilms produced.

As described above, depending on the concentration of nutrients, the structure of *L. monocytogenes* biofilm changed from multilayered cells in a rich medium BHI to a knitted structure formed by filaments and microcolonies in a poor medium BHI/10. Such different structures have been previously described in the literature (Rieu et al., 2008), although some studies described them as honeycomb (Borucki et al., 2003; Chavant et al., 2004; Guilbaud et al., 2015), which has similarities with a knitted network given the presence of holes and microcolonies but is different regarding the presence of filaments.

In another study, structural differences were also reported in some *L. monocytogenes* strains. Authors observed honeycomb and multilayered cell structures and suggested flagella as having a role in its formation since in the absence of the latter, they observed a homogenous multilayered cell structure instead of a honeycomb structure in static conditions (Guilbaud et al., 2015). This work have demonstrated, using a microfluidic system, that the concentration of nutrients alone affects the structure of *L. monocytogenes* biofilm.

In static conditions, homogeneous multilayered structures were observed and the biofilm architecture was completely different from the one observed in the microfluidic growth chamber. There were no structural differences between the rich and poor medium although the biofilm grown under poor medium was so low the biomass could be hardly seen in the 3D image analyses after size reduction. It is known that growth conditions affect biofilm morphology via differential gene expression systems like the quorum sensing *agr* system (Rieu et al., 2008). Indeed, it has been shown that in static conditions, *agr* system expression is barely detectable and the biofilm is organized in a multilayered cellular structure, while when grown in flow cells, biofilm is structured by a knitted network-like structures and an increased expression of the *agr* system over time could be noticed (Rieu et al., 2008). This could be the reason why the structure in static and microfluidic conditions was different. Furthermore, in a previous study, the *agr* system was shown to have an important role in biofilm structure in *S. aureus* (Periasamy et al., 2012). The filaments observed in our study are in accordance with the ones described in the work of Rieu et al. (2008). This difference seen in the structures could be due to the method used for biofilm formation; a knitted structure has been observed only in flowing conditions (Monk et al., 2004; Rieu et al., 2008; Renier et al., 2014) while the honeycomb structure has been described only in static conditions (Djordjevic et al., 2002; Marsh et al., 2003; Guilbaud et al., 2015). Long chains of *L. monocytogenes* cells were reported in previous studies (Bereksi et al., 2002; Monk et al., 2004; Giotis et al., 2007). According to these authors, these filamentous structures are produced when the bacterium is exposed to a range of stress conditions (Giotis et al., 2007) like high concentration of NaCl, presence or absence of acid (Bereksi et al., 2002), or limited nutrients (Monk et al., 2004). In our study, these filaments were clearly observed in rich medium and they were carried off by the flow. Shear stress could be involved in this morphology as it was not observed in our static study and anyone else's, to the best of our knowledge. It is still not understood which mechanism is involved in the formation of this cellular morphology, though a recent study showed that filament formation in *L. monocytogenes* is associated with a reduced secretion of two cell wall hydrolases (Renier et al., 2014). It is known that some bacteria, like *Myxococcus xanthus*, adopt a multicellular morphology in response to starvation (O'connor and Zusman, 1991; McBride and Zusman, 1993; Shimkets, 1999) and during antibiotic exposure (Justice et al., 2008). This morphology change is achieved through suppression of cell division in response to exogenous stresses. It has been suggested that filamentation is an adaptive response used by bacteria to increase survival under these hard growth condition (Justice et al., 2008).

Biovolume, as calculated after the DNA staining with Propidium Iodide and Syto9, showed that there is more biofilm in poor medium than in rich medium in microfluidic conditions, while in static condition there was more biofilm in rich medium than in poor medium. These contradictory results could be due to the fact that in static condition there is no renewal nutrients, an important limited factor, especially in poor medium, which could lead to the high decrease of the growth rate since in the poor medium, the overall biomass was dead or stressed. Moreover, inconsistent results were observed between biofilm formation in rich and poor medium under static condition in previous work, indeed while some studies showed that the poor medium enhance biofilm formation (Zhou et al., 2012; Combrouse et al., 2013; Kadam et al., 2013), others showed the contrary (Stepanovic et al., 2004), the nature of the medium and supplements used in these different studies could be the reason of the discrepancies observed.

Interestingly, the amount of dead biomass (and live biomass) is significantly higher in diluted medium than in rich medium under microfluidic condition but not in static condition which could be due to the high stress effect that bacteria faced in the absence of renewal nutrients as cited above. Our results in dynamic conditions confirm those of a previous study where it was observed that in poor medium the amount of dead biomass is higher than in rich medium (Kadam et al., 2013). In *P. aeruginosa*, it has been proposed that cell death plays an important role in biofilm development and in the dispersal of a subpopulation of surviving biofilm cells (Mai-Prochnow et al., 2004; Bayles, 2007; Allocati et al., 2015). Cell death and more specifically, cell lysis involves prophage induction in this species (Webb et al., 2003; Barraud et al., 2006). It is interesting to note that releasing genomic DNA is necessary for early attachment to glass and for biofilm formation in *L. monocytogenes* (Harmsen et al., 2010). Interestingly, in this bacterium, the extracellular matrix is composed essentially of e-DNA and proteins (Combrouse et al., 2013); this could be the reason why after treatment with DNase the biofilm grown in a poor medium detached within seconds (Figure 6A). In other studies it was reported that e-DNA also has a structural role, acting as an interconnector compound and stabilizing biofilm structure in *P. aeruginosa* (Allesen-Holm et al., 2006; Flemming et al., 2007; Yang et al., 2007; Das et al., 2010) and in *S. aureus* (Qin et al., 2007; Rice et al., 2007; Izano et al., 2008). These conclusions are consistent with our findings with *L. monocytogenes* biofilm. As this study confirmed here, e-DNA, present under limited nutrient conditions, plays an important role in the knitted network-like structure since the filaments completely disappeared after treatment with DNase I and the biomass grown under this condition showed biofilm made up of single cells (Figure 6). In a recent study, Zetzmann et al., 2015 explained that the presence of more e-DNA in a limited-nutrient medium could be due to the hypotonic condition that leads to cell lysis and thus the release of genomic DNA (Zetzmann et al., 2015). The hypotonic effect may contribute to the presence of e-DNA via cell lysis, but, since in our study there was not an increase in dead biomass in a limited nutrient compared to a rich medium under static conditions, it is difficult to explain the presence of more dead cells through the hypotonic effect only.

In static conditions, there was more biofilm and more dead biomass in the rich medium than in the poor medium. Our results are not consistent with previous observations where in the poor medium there were more dead cells than in a rich medium under the same conditions for biofilm formation (Kadam et al., 2013). In our study, the amount of dead biomass was evaluated by calculating biovolume; in the previous study, researchers used nutrient broth (NB) as a poor medium whereas in our study a 10-fold diluted BHI medium was used. Maybe the different composition of the two media, especially the presence of glucose in NB which enhances biofilm formation as observed in *Burkholderia pseudomalei* (Ramli et al., 2012), and nutrient limitation can affect biomass production.

In the present study, biofilm was studied with two strains—Lm76 and Lm132 from 1/2a and 1/2b serotypes respectively. Serotype 1/2a belongs to lineage II and is commonly isolated from foods, but also isolated in human listeriosis, whereas 1/2b belongs to lineage I and is commonly isolated in human listeriosis cases (Gray et al., 2004; Van Stelten and Nightingale, 2008; Van Stelten et al., 2011; Cruz et al., 2014). Statistical analysis showed no significant differences between biovolume of Lm76 and Lm132 in both static and microfluidic conditions. In our preliminary results these two strains were classified as strains with high biofilm formation capacity (data not shown). In previous studies, there have been discrepancies regarding biofilm formation results between lineage I and II (Djordjevic et al., 2002; Borucki et al., 2003; Combrouse et al., 2013). Some of these studies showed that strains from lineage I formed better biofilm than lineage II (Djordjevic et al., 2002; Takahashi et al., 2009) whereas other studies reported that strains from lineage I form less biofilm than lineage II (Norwood and Gilmour, 2001; Borucki et al., 2003; Combrouse et al., 2013). The culture conditions (temperature, medium) used in these different studies might be the underlying cause for the divergent results, as suggested previously (Combrouse et al., 2013). Since *L. monocytogenes* biofilm gives a great cause of concern, it is important to study this biofilm in the same environmental conditions of food industries as possible. The growth temperature used in this work and in many other studies (Djordjevic et al., 2002; Rieu et al., 2008; Combrouse et al., 2013) did not reflect that of the food industries, therefore, it is important to consider the low temperature growth in *L. monocytogenes* biofilm studies to provide a better interpretation in the context of food industries.

## Conclusion

This study showed that limited nutrients are associated with an increase of *L. monocytogenes* biofilm production when measured in dynamic conditions with controlled shear forces. Moreover, this study showed that limited nutrients promote *L. monocytogenes* biofilm structuration and that under low concentration of nutrients, *L. monocytogenes* population organizes a combination of filaments and microcolonies to form a stable knitted network-like structure in microfluidic condition. This study highlights the link between volume of biofilm formation and the proportion of dead and damaged cells in the communities. Thus, this study showed in limited nutrient conditions, the presence of e-DNA associated with cell death enhances biofilm development and stabilizes the structure in microfluidic conditions but not in static conditions. These results highlight the need to identify processes that specifically target e-DNA during sanitation procedures to prevent biofilm formation and thus preventing food contamination by *L. monocytogenes* in food processing facilities.

## Author contributions

TC and PF conceived the project, TC performed the experiment. TC analyzed the data. TC, PF, MJ, and SQ interpreted the results. All authors contributed to the experimental design and have read and approved the manuscript.

## Funding

This work is supported by the Natural Science and Engineering Research Council of Canada N°RDC: PJ 445805-12.

### Conflict of interest statement

The authors declare that the research was conducted in the absence of any commercial or financial relationships that could be construed as a potential conflict of interest.

## Supplementary material

Video S1 **Time lapse phase-contrast microscopy was performed across 24 h with 16 frames per seconds to show ***Listeria monocytogenes*** biofilm grown under BHI medium in microfluidic system**. The video shows filaments detached from the chamber surface under the flow effect.

Video S2 **Time lapse phase-contrast microscopy was performed across 24 h with 16 frames per seconds to show ***Listeria monocytogenes*** biofilm grown under BHI (the top section) and BHI/10 (The bottom section)**.

Video S3 **Time lapse phase-contrast microscopy was performed across 18 h with 16 frames per seconds to show ***Listeria monocytogenes*** biofilm grown under BHI (in the top section) and BHI/10 (in the bottom section) supplemented with DNase I at the final concentration of 100 μg/ml**.

Video S4 **Time lapse phase-contrast microscopy was performed across 18 h with 16 frames per seconds to show ***Listeria monocytogenes*** biofilm grown under BHI/10 without DNase I treatment (in the top section) and BHI/10 (in the bottom section) supplemented with DNase I at the final concentration of 100 μg/ml**.
